# Rethinking Vasopressin: New Insights into Vasopressin Signaling and Its Implications

**DOI:** 10.34067/KID.0000000000000194

**Published:** 2023-06-26

**Authors:** Jason A. Watts, Juan Pablo Arroyo

**Affiliations:** 1 Epigenetics and Stem Cell Laboratory, National Institute of Environmental Health Sciences, National Institutes of Health, Research Triangle Park, North Carolina; 2 Division of Nephrology and Hypertension, Department of Medicine, Vanderbilt University Medical Center, Nashville, Tennessee; 3 Vanderbilt Center for Kidney Disease, Vanderbilt University Medical Center, Nashville, Tennessee

**Keywords:** vasopressin, aquaporin 2, antidiuretic hormone, AVP, metabolic syndrome, CKD

## Abstract

Vasopressin is a highly conserved peptide hormone that has been traditionally associated with water homeostasis. There is accumulating evidence in both humans and animal models that vasopressin is implicated in the regulation of metabolism. This review focuses on the effects that vasopressin exerts on the regulation of glucose and fatty acids with a particular emphasis on the potential repercussions of metabolic dysregulation in kidney disease.

## Introduction

**Figure 1.**
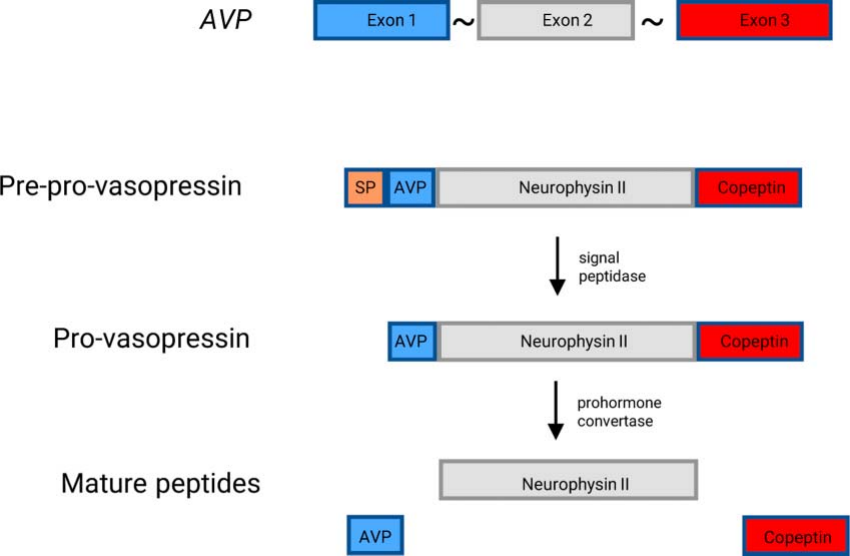
**Gene processing of vasopressin and resulting peptides.** Pre-pro-vasopressin is cleaved by signal peptidase to release the SP. Pro-vasopressin is processed by prohormone convertase to release AVP. AVP, arginine-vasopressin; SP, signal peptide.

Vasopressin is an evolutionary conserved peptide with pleiotropic functions whose origins can be traced back over 600 million years.^1^ Vasopressin, or its homologue vasotocin, is found in organisms as wide-ranging as arthropods, nematodes, sea squirts, and mammals. This degree of evolutionary conservation suggests that vasopressin plays a critical role in regulating physiologic processes.^2^ Despite the diverse organisms that express vasopressin, its functions converge on three main categories: metabolic regulation, reproduction, and the maintenance of salt and water homeostasis.^1–4^ Vasopressin is synthesized as a large 164 amino acid pre-pro-peptide, which is processed and cleaved to produce three distinct peptides: the biologically active vasopressin hormone, neurophysin-2, and the carboxy-terminus peptide copeptin (Figure 1). Vasopressin exerts its biologic effects through binding to three different G-coupled protein receptors: V1aR, V1bR, or V2R.

**Figure 2.**
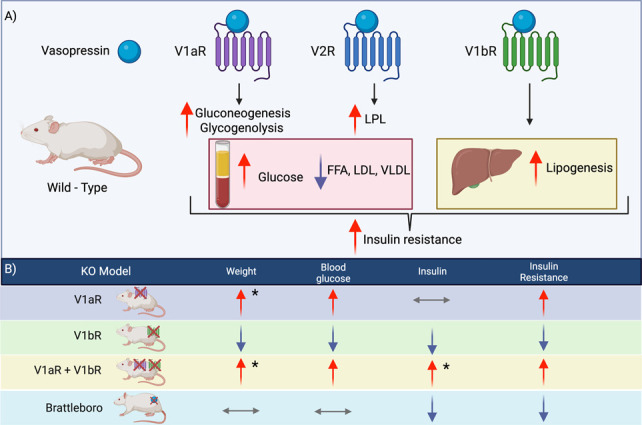
**Vasopressin receptors regulate glucose and fat metabolism.** In wild-type animals, vasopressin increases gluconeogenesis, glycogenolysis, increases lipoprotein lipase (LPL) activity, and increases hepatic lipogenesis (A). Phenotypic differences in metabolic parameters between vasopressin pathway knockout animal models (B). *When fed a high-fat diet.^15,17–23^ Created with BioRender. FFA, free fatty acids.

Vasopressin is known for its role in the regulation of water balance and BP. Vasopressin increases water reabsorption in the kidney *via* V2R and induces vasoconstriction *via* V1aR (See reviews^5–8^). However, its appearance in evolution before the presence of terrestrial life points to the importance of its role in processes beyond water conservation and BP control in land animals. Recent evidence has highlighted the involvement of vasopressin in the regulation of metabolism, where it has been shown to play a role in carbohydrate and lipid metabolism.^9–16^ Dysregulation of vasopressin signaling has been implicated in the development of metabolic disorders, such as diabetes and obesity. In this review, we will focus on the effect of vasopressin on the metabolism of glucose and lipids (Figure 2).

## Vasopressin and Metabolic Disease in Humans

There is accumulating genetic and functional evidence that vasopressin is a metabolic regulator in humans. Population-based studies have revealed multiple single-nucleotide polymorphisms in the vasopressin gene pathway (*AVP*, *AVPr1a*, and *AVPr1b* genes) that are associated with hyperglycemia, insulin resistance, elevated body mass, and diabetes mellitus.^9,24,25^ Vasopressin is difficult to measure in blood samples because of its low concentration, small size, and rapid degradation. Copeptin is produced at a 1:1 ratio with vasopressin (Figure 1) and is more stable in plasma samples, and therefore, it is used as a surrogate for vasopressin levels.^15,26–28^ Multiple studies have found an association between elevated blood copeptin levels, high-fat diet, metabolic syndrome, cardiovascular disease, diabetes mellitus, and CKD.^29–32^ Vasopressin is made in the hypothalamus and released by the posterior pituitary. Traditional stimuli for the release of vasopressin include hypotension and increased serum osmolality.^33^ Multiple additional stimuli that are associated to vasopressin release are also linked to metabolic derangements. In fact, Robertson’s group showed that vasopressin release was altered in patients with diabetes.^10,34–36^ In adults aged 51–70 years, underhydration was associated with obesity, insulin resistance, diabetes, low HDL, and metabolic syndrome.^37^ Moreover, in a Dutch study of outpatients with diabetes mellitus, higher copeptin levels were associated with both higher cardiovascular and all-cause mortality.^38^ Similarly in an American cohort of nondiabetic patients with CKD, copeptin was associated with insulin resistance.^39^ These results implicate the vasopressin system as a metabolic regulator in humans.

Although there are numerous studies in both human and animal models that show that the vasopressin system can directly modulate metabolism, there is no unified mechanistic understanding of how the vasopressin system regulates metabolism because its effects are frequently context dependent. Therefore, we will summarize specific observations of how the vasopressin system can regulate glucose and lipid metabolism (in-depth reviews of vasopressin and metabolism have been published previously).^15,16,40^ Patients with diabetes have elevated vasopressin levels, and this was thought to be a consequence of increased plasma osmolality due to hyperglycemia. However, in response to saline infusion, Zerbe *et al.* and Iwasaki *et al.* found that patients with diabetes mellitus had higher levels of vasopressin than could not be explained by the change in serum osmolality suggesting that there are nonosmotic mechanisms involved in the production of vasopressin.^10,36,41^ Data supporting nonosmotic regulation of vasopressin synthesis come from the observation that a glucose infusion in nondiabetic patients lead to reduced vasopressin levels despite an increase in plasma osmolality.^34,35^ In patients with diabetes, the infusion of vasopressin increased circulating glucose levels by promoting glycogen breakdown and gluconeogenesis.^29^ Together, these observations are consistent with a negative feedback loop between vasopressin and blood glucose, where vasopressin stimulates increased serum glucose but hyperglycemia decreases vasopressin.

The relationship between vasopressin and glucose level may be explained by the effect of vasopressin on glucose-regulating hormones. Enhorning and colleagues showed that in healthy patients with a high baseline copeptin and concentrated urine, both acute (1 hour) and chronic (1 week) water loading led to a significant decrease in glucagon.^42^ Data from the Korea National Health and Nutrition Examination Survey showed that low hydration status was linked to insulin resistance and changes in fat distribution.^43^ These data highlight the interaction between the regulation of glucose and vasopressin signaling.

Less data exist regarding the effects of vasopressin on fatty acid metabolism in humans. In healthy patients, treatment with a V1aR agonist (lysine-vasopressin) led to a decrease of nonesterified fatty acids in plasma.^44^ In addition, patients with central diabetes insipidus treated with a V2R agonist (desmopressin) led to an increase in lipoprotein lipase activity and a decrease in both LDL and VLDL^45^ while the use of a V2R antagonist (tolvaptan) in patients with polycystic kidney disease has been reported to increase LDL and total cholesterol.^46^ Together, the association and experimental data in humans strongly suggest that vasopressin is involved in regulating both glucose and lipid metabolism. In the next section, we will review the animal models in which the regulation of glucose and lipids by vasopressin has been studied.

## Vasopressin and Glucose Metabolism

Vasopressin has been shown to alter blood glucose level, tissue-specific uptake of glucose, and the intracellular fate of glucose after it is taken up by the cell. Vasopressin administration can increase blood glucose levels in both humans and animals by increasing hepatic gluconeogenesis and glycogenolysis (Figure 2).^13,17,47,48^ In a rat model, the hyperglycemic effect of vasopressin infusion by mini-pump was attenuated by blocking V1aR but not V1bR, suggesting that vasopressin-induced hyperglycemia is mediated, at least in part, through the V1a receptor.^17^ However, the V1aR knockout mouse had higher plasma glucose at baseline, was insulin resistant, and was prone to obesity.^18^ These seemingly contradictory results, where loss of signaling through the V1a receptor can either increase or decrease serum glucose, highlight the link between V1aR signaling and glucose homeostasis as well as our incomplete understanding of the role of vasopression in glycemic control.

Vasopressin can also modify insulin sensitivity and resistance and thus alter tissue-specific glucose uptake. Activation of insulin signaling and glucose uptake by cells can be monitored using AKT phosphorylation as a marker of insulin activity. V1aR knockout mice have decreased AKT phosphorylation in adipocytes in vitro, which suggests that V1a expression and signaling can modify insulin sensitivity.^19^ Using a V1b receptor knockout mouse model, Oshikawa *et al.* showed that vasopressin can stimulate insulin release from pancreatic islet cells.^49^ However, further studies showed that despite the inability to stimulate insulin release from pancreatic islet cells, V1bR knockout mice had lower glucose levels and increased insulin sensitivity. In these mice, AKT phosphorylation was increased in white adipose tissue suggesting that loss of V1b receptor activity improved insulin sensitivity in adipocytes. These effects are potentially due to a concomitant decrease in glucagon and a net increase in the insulin:glucagon ratio.^20^ To clarify the role of V1a and V1b receptors in glucose homeostasis, Nakamura *et al.* generated a double V1a and V1b receptor knockout mouse. The V1aR and V1bR double knockout mouse had insulin resistance, high plasma glucose, and increased body weight while on a high-fat diet.^15,21^ These data suggest that both V1aR and V1bR are involved in glucose homeostasis, and a critical balance of V1aR and V1bR activation may exist.

Once the glucose is inside cells, there are data that suggest that vasopressin can regulate its metabolic fate. In isolated hepatocytes, stimulation with vasopressin led to increased conversion of pyruvate to lactate.^50,51^ In starved rat hepatocytes, vasopressin stimulated gluconeogenesis by activating oxoglutarate dehydrogenase, an effect that is dependent on intracellular calcium levels.^52^ The in vivo effects of vasopressin flux in the cells are unclear. However, the work by Johnson *et al.* has shown that vasopressin plays a key role in the regulation of fructose metabolism and ultimately metabolic health.^11,53,54^ This suggests that vasopressin can regulate carbohydrate metabolism beyond glucose.

In addition to vasopressin regulating insulin signaling, insulin itself can regulate vasopressin levels. Keller *et al.* identified a protease, the insulin-regulated aminopeptidase (IRAP), that can cleave vasopressin both in vivo and in vitro. IRAP is a zinc metalloprotease that is found in the same vesicles as the glucose channel GLUT4 in muscle and adipose tissue. Similar to what happens with GLUT4, insulin signaling increases membrane expression of IRAP which then leads to the proteolytic cleavage of vasopressin.^55–58^ The IRAP knockout mice have increased circulating vasopressin levels, insulin resistance, and persistent urinary concentration.^56,59^ Whether there is a direct link between IRAP, insulin resistance, high blood vasopressin levels, and cardiovascular disease is still unknown.

The exact mechanisms through which vasopressin regulates insulin signaling, blood glucose levels, and the metabolic fate of glucose are still unclear. As discussed above, the effect of vasopressin on insulin signaling and glucose levels are context dependent and can be modified by the activation and/or inhibition of the V1a and V1b receptors. Moreover, there is evidence showing that vasopressin can regulate secretion of adrenocorticotropic hormone (ACTH) leading to higher cortisol levels, which in turn contribute to hyperglycemia and insulin resistance.^60–62^ Additional research to understand the physiologic and pathologic conditions in which vasopressin's effects take place will be necessary to understand what currently seem to be contradictory effects by the same hormone.

## Vasopressin and Lipid Metabolism

Vasopressin is a known regulator of lipid metabolism. Observations by Bergen *et al.* and Mirsky and Linn in the 1960s showed that in addition to the hyperglycemic effects mentioned above, the injection of vasopressin or oxytocin to nondiabetic and alloxan-diabetic dogs caused a significant decrease in plasma-free fatty acids, and the continuous infusion of vasopressin had a larger effect than oxytocin.^48,63^ These observations were followed by experiments that showed in starved animals, vasopressin decreased circulating ketone bodies.^64,65^ These effects were shown to be independent of increases in glucose or insulin as the infusion of epinephrine led to increased plasma insulin with minimal changes to ketone levels. However, it was unclear whether the effect was due directly to vasopressin or some secondary cause given that the antilipolytic effects of vasopressin in vivo were difficult to replicate in vitro.^65^ Further research now suggests that, similar to glucose, vasopressin can have both antilipolytic and lipolytic effects.^16^

Receptor specific effects of vasopressin on lipid metabolism were elucidated with the use of transgenic models (Figure 2). Hiroyama *et al.* showed that mice lacking the V1a receptor had high blood glycerol and ketone bodies and low free fatty acids, in conjunction with high carnitine and acylcarnitines, suggesting ongoing lipolysis and beta-oxidation in vivo.^19^ *In vitro* analysis of brown adipose tissue from the V1a receptor knockout mice showed that knockout mouse adipocytes were more sensitive to isoproterenol induced lipolysis, supporting a direct effect of V1aR stimulation on lipid synthesis. Why the V1aR knockout mice have increased rates of lipolysis and can become obese on a high-fat diet is still unclear. The same group studied the role of the V1b receptor in lipid metabolism in V1b receptor knockout animals.^20,22^ They showed that V1bR knockout mice have the opposite phenotype of the V1aR knockout animals: low body weight, low blood glycerol levels, and suppressed beta-oxidation. Moreover, in vitro assays on isolated adipocytes from V1bR knockout animals showed decreased sensitivity to isoproterenol-induced lipolysis.^21^ Recent experiments highlight the importance of V1b receptor signaling. In an elegant set of experiments with various transgenic mouse models, Andres-Hernando *et al.* showed that the V1b receptor, but not V1a receptor, was required for the development of fructose-induced metabolic syndrome.^11^ After treatment with fructose in the diet, the V1b receptor knockout mice had lower body weight as well as triglyceride, alanine aminotransferase, insulin, leptin levels, and liver steatosis compared with wild-type littermate controls. These effects were preventable with increased hydration, suggesting that vasopressin plays a key role in the development of fructose-induced metabolic syndrome. Interestingly, the V1a receptor knockout mice fed a high-fructose diet had a worse phenotype than wild-type littermate controls, thus supporting the key observation that vasopressin links fructose and lipid metabolism. Other groups have shown that vasopressin plays a role regulating parallel pathways that affect lipid metabolism including Uncoupling protein 1 expression in brown adipocytes, thermoregulation, and behavior.^66–70^ These results highlight the complex and incompletely understood interplay between hydration status, fatty acid metabolism, and vasopressin.

## Metabolism in the Brattleboro Rat

The Brattleboro rat has been the classic model to study the function of vasopressin.^71^ Brattleboro rats have a vasopressin pre-pro-peptide processing defect because of a single base pair deletion in the coding region of carrier protein neurophysin-2 (Figure 1), which leads to altered vasopressin processing and the absence of detectable circulating vasopressin.^72^ Brattleboro rats have diabetes insipidus and a number of other phenotypic characteristics, including low gestational weight, stunted growth, decreased ACTH, and decreased corticotropin-releasing hormone.^73,74^ These last results are consistent with reports that vasopressin increases ACTH abundance. Whether Brattleboro rats have altered metabolism has not been studied in depth. However, Nakamura *et al.* evaluated glucose tolerance in Brattleboro rats by giving the rats an oral glucose load. They found that at baseline Brattleboro rats have similar plasma glucose levels to controls but lower insulin levels, which suggest an increased sensitivity to insulin.^23^ They also found that Brattleboro rats had significantly lower AUC plasma insulin levels than controls, further confirming the increased sensitivity to insulin. These results are intriguing given the varied phenotypes of the V1a, V1b, and V1a/V1b receptor knockout mice. The authors suggest that vasopressin's role in glucose homeostasis is a result of an interplay between all vasopressin receptors (including V2R) and possibly oxytocin (which is elevated in Brattleboro rats).^23^

## Vasopressin and the Kidney

Beyond its effects on water reabsorption, vasopressin has been associated with worsening kidney disease.^39,60,75–78^ How much of these effects are due to altered water handling versus other signaling pathways is currently unclear. Higher levels of vasopressin along with increased V2R signaling have been implicated in worsening diabetic nephropathy.^78–80^ It is known that at least some of the deleterious effects of vasopressin on the progression of diabetic nephropathy are due to increases in the renin-angiotensin-aldosterone system and sympathetic activity. In patients with long-standing type 1 diabetes, higher copeptin levels were associated with higher intrarenal renin-angiotensin activity and progression of diabetic kidney disease.^81^ In fact a provocative study showed that in diabetic patients with severe fluid retention despite treatment with loop diuretics, the addition of a V2R antagonist (tolvaptan) resulted in significant diuresis and improved decongestion.^82^ This supports the notion that vasopressin plays a key role in volume retention associated with diabetic nephropathy. In addition, there are data that suggest that the interaction between vasopressin, insulin, and glucose plays a key role in the progression of kidney disease. We found that copeptin levels increase in patients with CKD who also have peripheral insulin resistance, but not hepatic insulin resistance, suggesting a multilayered regulation pathway.^39^ Nakagawa *et al.* have suggested that the interaction between fructose metabolism, vasopressin, and the polyol pathway could be playing a role in the protective role of sodium-glucose cotransporter-2 inhibitors in progression of CKD.^83^ One of the potential mechanisms for progression of kidney disease is related to high-protein diet–induced hyperfiltration; interestingly, both vasopressin and glucagon are required for the hyperfiltration response.^60^ Together, these observations suggest that there are still a number of unanswered questions regarding the biologic activity of vasopressin that are not addressed with our current physiological model of vasopressin function. Recently, we reported that kidney tubular epithelial cells make functional vasopressin.^84^ The physiologic relevance of this observation remains to be ascertained. However, if there is functionally relevant tissue-specific production of vasopressin, this could be an exciting new area of research that could add to the remarkable body of work on what is a relatively simple nine amino acid peptide that has been around for over 600 million years.

## Perspectives and Conclusion

It is now widely accepted that vasopressin is a key regulator of metabolic pathways involving carbohydrate and lipid metabolism. The field of non-antidiuretic actions of vasopressin has been advanced significantly over the past few years^9,11,14,15,24,25,30,32,42,53,54,60,78,85^; however the specific feedback loops through which this system is regulated remain to be clarified. The multiplicity of observations in which vasopressin can have opposite effects *i.e.*, increase and decrease blood glucose and increase and decrease lipid synthesis suggest that there are mechanisms and feedback loops yet to be discovered. Therefore, the study of vasopressin has been, and continues to be, an exciting area of research that promises to have a significant impact on our understanding of health and disease.
